# The bibenzyl derivatives of *Dendrobium officinale* prevent UV-B irradiation induced photoaging via SIRT3

**DOI:** 10.1007/s13659-022-00323-6

**Published:** 2022-01-27

**Authors:** Ding-kang Chen, Hui-yan Shao, Liu Yang, Jiang-miao Hu

**Affiliations:** 1 State Key Laboratory of Phytochemistry and Plant Resources in West China, Kunming Institute of Botany, Chinese Academy of Sciences, Kunming, 650201 China; 2 R&D Center of Dr. Plant, Kunming Institute of Botany, Chinese Academy of Sciences, Kunming, 650201 China; 3 University of Chinese Academy of Science, Beijing, 100049 China

**Keywords:** *Dendrobium officinale*, Bibenzyl derivatives, Skin photoaging, Oxidative stress, SIRT3

## Abstract

*Dendrobium officinale* is a valuable medicinal herb that is widely used in traditional Chinese medicine. The chemical constituents of *D. officinale* have attracted much attention and a large number of compounds have been reported including many bibenzyl derivatives. 13 bibenzyl derivatives from *D. officinale* were sent for molecular docking, surface plasmon resonance (SPR) assay and after detection of Mn-SOD and SIRT3 activities in or not in HaCaT cells, it was concluded that bibenzyl derivatives did not directly activate Mn-SOD but promoted SIRT3 proteins. In addition, HaCaT cells were irradiated with UV-B to induce an oxidative stress model in vitro to further verify the effect of bibenzyl derivatives. The results show that bibenzyl derivatives could directly bind to SIRT3, enhance the deacetylation and then activate Mn-SOD, so as to protect UV-B induced skin photoaging.

**Graphical Abstract:**

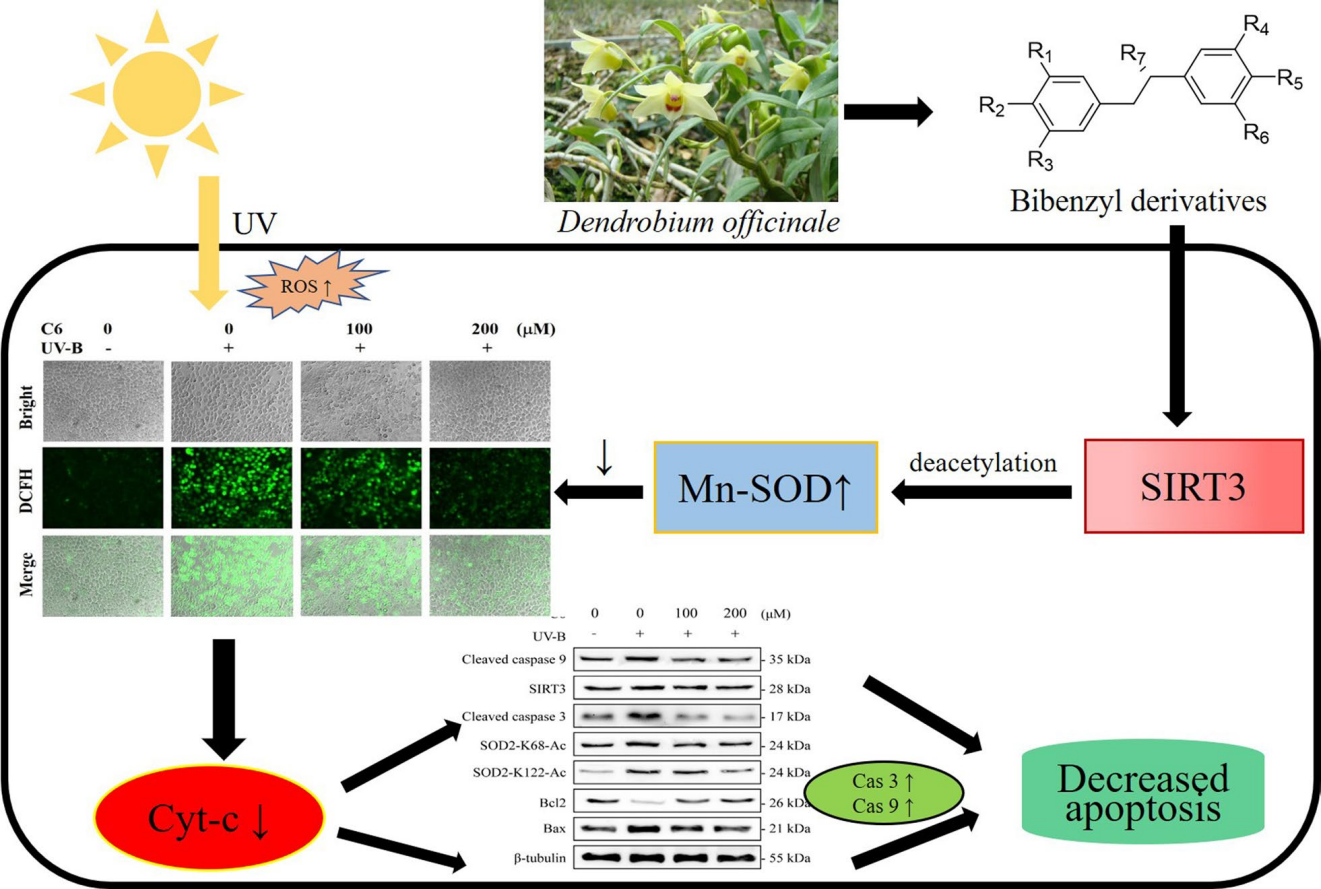

## Introduction

Skin photoaging is mainly caused by long-term contact with ultraviolet (UV)-A (400–320 nm) and UV-B (320–280 nm) radiation in sunlight, resulting in skin morphological changes [1]. Mild photoaging is characterized by aging phenomena such as wrinkles and laxities of the skin, which affects the beauty and appearance and severe photoaging even will cause a variety of skin diseases and even tumors [2]. The unified pathogenic factor of these changes is high level of reactive oxygen species (ROS) in skin cells caused by UV-B irradiation [3]. Clinically, the symptoms of skin photoaging are generally treated with chemically synthesized products (such as sunscreen, salicylates, *O*-aminobenzoates, etc.) and usually accompany serious skin diseases especially for sensitive people with long term use of these chemical synthetic products [4]. Therefore, it is urgent to find a more effective and security method for the prevention and treatment of skin photoaging.

Manganese superoxide dismutase (Mn-SOD) is a major superoxide anion scavenging enzyme in the mitochondrial matrix [5]. It reduces mitochondrial oxidative stress by converting superoxide anion into hydrogen peroxide. Sirtuins (SIRT 1–7) are a series of proteins with deacylase activity which change activity of substrate proteins by removing the acetyl group of the target protein, so as to regulate life activities [6]. SIRT3 is a major mitochondrial deacetylase, which plays an important role in regulating mitochondrial metabolism and energy production [7]. Mn-SOD activity is regulated by SIRT3 mediated deacetylation. Mn-SOD in mitochondrial matrix is the substrate of SIRT3 deacetylase. Deacetylation at K68 and K122 sites enhances the Mn-SOD activity, so reduces the damage caused by oxidative stress [8, 9]. These results suggest that the deacetylation activity of SIRT3 plays an important role in maintaining redox homeostasis.

*Dendrobium officinale* is a valuable medicinal herb that is widely used in traditional Chinese medicine. *D. officinale* has a wide range of pharmacological applications, which is due to it containing a variety of chemical substances, including polysaccharides, stilbenoids and their derivatives, lignans [10, 11]. Our research group has long been committed to the study of chemical composition and activity application of *Dendrobium*. In previous studies, we analyzed the structure of polysaccharides extracted from *D. officinale* and revealed that polysaccharides can reduce blood glucose by promoting the secretion of glucagon-like peptide-1 [12]. Besides, by detecting the free radical scavenging ability, tyrosinase inhibition ability and the ability to promote the production of collagen, bibenzyl derivatives in *Dendrobium* may be candidates for antioxidants, skin whitening and anti-aging agents [13]. Recent works have shown that the dried stems of *D. officinale* are used in the treatment of inflammatory [14], diabetes [15], and immunomodulatory [16]. Furthermore, *D. officinale* has the functions of moisturizing and anti-aging activities, as recorded in “the secret prescriptions of Waitai”, “the prescriptions of Taiping Huimin Heji”, and “the prescriptions of Puji” [17]. There are works have shown that that taking orally of *D. officinale* juice in aging mice induced by D-galactose caused anti-aging effect, and the contents of SOD and glutathione peroxidase in serum and organs of mice increased significantly [18]. Meanwhile, *D. officinale* protocorm treatments externally can protect the skin from photoaging by increasing the expression levels of CAT and SOD [19].

Bibenzyl derivatives are the major active compound which are abundant in *D. officinale*, has exhibited various pharmacological effects including antioxidant activity in vitro, but the potential molecular mechanism is still unclear [17]. Since SIRT3 protein plays an important role in maintaining redox homeostasis in vivo, we speculate that bibenzyl derivatives can inhibit skin photoaging by activating SIRT3 protein. In this study, we tested this hypothesis using HaCaT immortalized human keratinocytes in vitro.

## Results

### Bibenzyl derivatives binds to SIRT3 protein

**Fig. 1:**
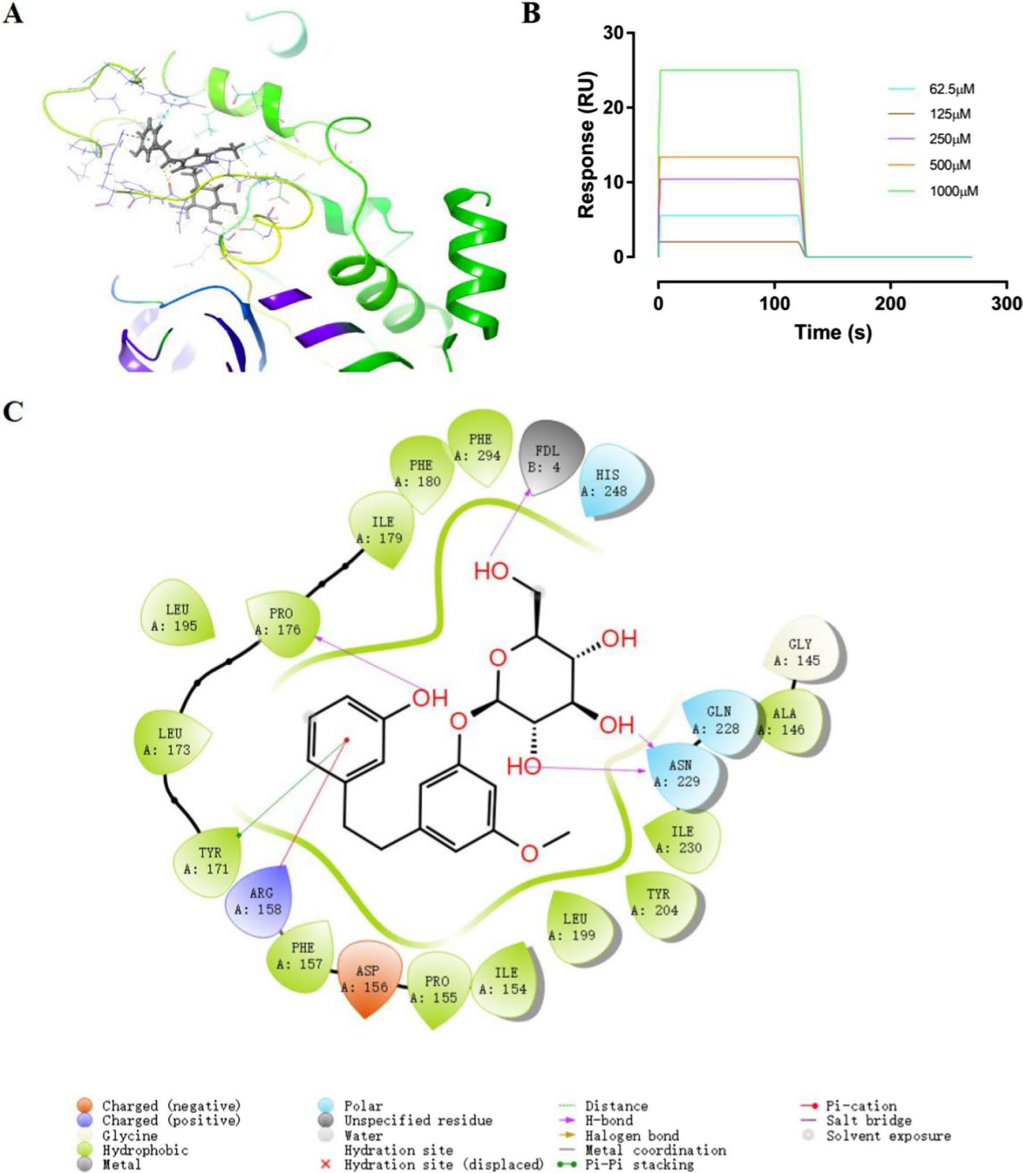
Interactions between bibenzyl derivatives in *D. officinale* and SIRT3 protein. The following is an example of compound **6** (C6). **A** C6 bound to SIRT3 protein at a suitable binding pocket predicted by molecular docking. **B** Sensorgram of C6 at various concentrations (62.5, 125, 250, 500 and 1000 μM) binding to SIRT3 protein. **C** Receptor-based molecular docking protein–ligands interactions profile of SIRT3 protein with C6

SIRT3 is a major mitochondrial NAD^+^ dependent deacetylase, which plays an important role in regulating mitochondrial metabolism and energy production, and is related to the beneficial effects of exercise and caloric restriction. SIRT3 has gradually become a potential therapeutic target for the treatment of metabolic and neurological diseases [20]. Recently, molecular docking study of SIRT3 protein against 13 bibenzyl derivatives in *D. officinale* was carried out using receptor based molecular docking by Maestro 11.9. The GScore data obtained from molecular docking of 13 bibenzyl derivatives are illustrated in Table 1. All bibenzyl derivatives show a degree of binding cooperation with SIRT3 protein (GScore < − 6). In the present study, compound **6** (C6) get a best GScore (− 9.821) for SIRT3 protein receptor compared to other bibenzyl derivatives. The most favorable binding site was identified by a comparison of the free binding energies at various binding sites (Fig. 1A). The protein-ligands interactions yielded a large of information including lipophilic, salt bridges, electrostatic and hydrogen bonding interaction. For instance, Fig. 1C shows the frequency of the interactions between C6 with the receptor during the simulation. A main portion of the interactions are formed via hydrogen bonds with residues namely ASN229, FDL4 and PRO176. Interaction of π–cation is noted to form with ARG158, as well as π–π stacking with TYR171. It is speculated that due to the introduction of glucose group, there are more hydrogen bonds between compound and SIRT3 protein, so that C6 performs better in molecular docking assay.

**Table 1:** Different GScores in receptor based on molecular docking and K_D_ data based on SPR method of bibenzyl derivatives in *D. officinale* involved in the protein–ligands interaction

Compounds no.	GScore	K_D_ (M)
**1**	− 7.211	9.686 × 10^−4^
**2**	− 7.150	1.085 × 10^−3^
**3**	− 7.607	3.611 × 10^−4^
**4**	− 7.638	1.059
**5**	− 6.600	3.465
**6**	− 9.821	2.164 × 10^−3^
**7**	− 7.570	7.065 × 10^−4^
**8**	− 7.627	3.115 × 10^−4^
**9**	− 6.094	0.2010
**10**	− 8.385	0.5223
**11**	− 7.496	1.423 × 10^−3^
**12**	− 6.253	7.158 × 10^−4^
**13**	− 6.731	5.578 × 10^−4^

To further study whether bibenzyl derivatives in *D. officinale* directly interacts with SIRT3 protein, we evaluated their binding affinity using a surface plasma resonance (SPR) system. Consistent with the sensorgram of bibenzyl derivatives at various concentrations (62.5–1000 μM, double dilution), the binding parameter of dissociation rate contents (K_D_) were obtained (Fig. 1B). The SPR analysis showed that bibenzyl derivatives had a varying degree of binding directly to SIRT3 protein with a K_D_ range of 3.115 × 10^−4^ (M) to 3.465 (M) (Table 1).

### Bibenzyl derivatives increase cell viability

**Fig. 2:**
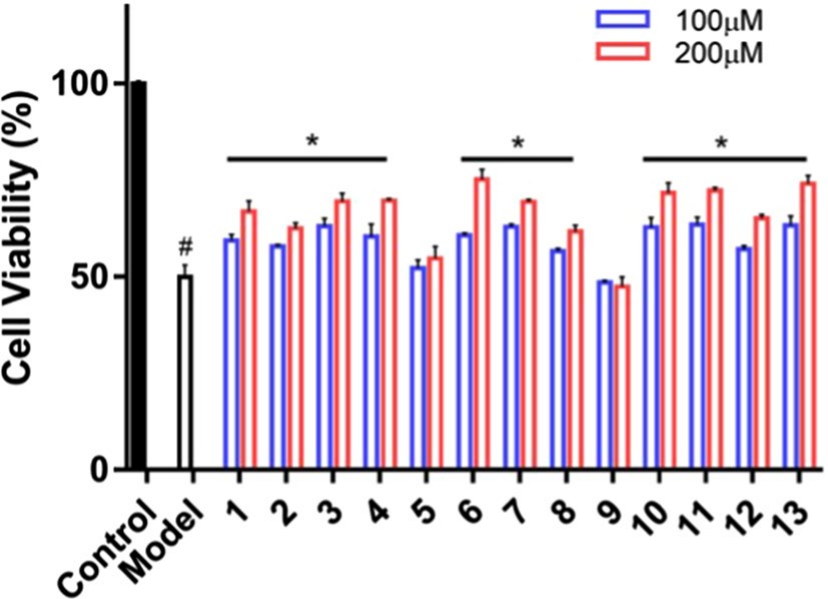
Bibenzyl derivatives in *D. officinale* improve the survival rate of cells. ^#^P < 0.05, significantly different from control group; *P < 0.05, significantly different from model group

UV-B irradiation has been known to induces oxidative stress, leading to the accumulation of reactive oxygen species (ROS) and reducing cellular antioxidant capacity in skin cells [21]. Excessive UV-B irradiation will not only affect the appearance of the skin and produce aging manifestations such as sunburn, redness and swelling, but also induce other skin related diseases and even tumors [22]. In order to further confirm the antioxidant effect of bibenzyl derivatives in *D. officinale* and its potential mechanism at the cell level, the protection of the bibenzyl derivatives on the HaCaT cells after UV-B irradiation was detected by MTS assay. The cell viability of HaCaT cells in the control group was considered as 100%. Compared with the control group, the cell viability of HaCaT cells in the model group after UV-B irradiation was significantly lower, indicating that the model was successfully established. Compared with the model group, the cell viability of cells treated with bibenzyl derivatives in *D. officinale* at the concentration of 100 and 200 μM increased significantly with a concentration-dependent manner, except for compounds **5** and **9** when both compounds did not perform well in the molecular docking and surface plasmon resonance measurement (Fig. 2).

For ease of discussion and in view of the amount of compound stored in the laboratory, we chose C6 as the representative in the subsequent experiments to explore the mechanism of anti-photoaging of bibenzyl derivatives in *D. officinale*.

### Bibenzyl derivatives reduces apoptosis in HaCaT cells

**Fig. 3:**
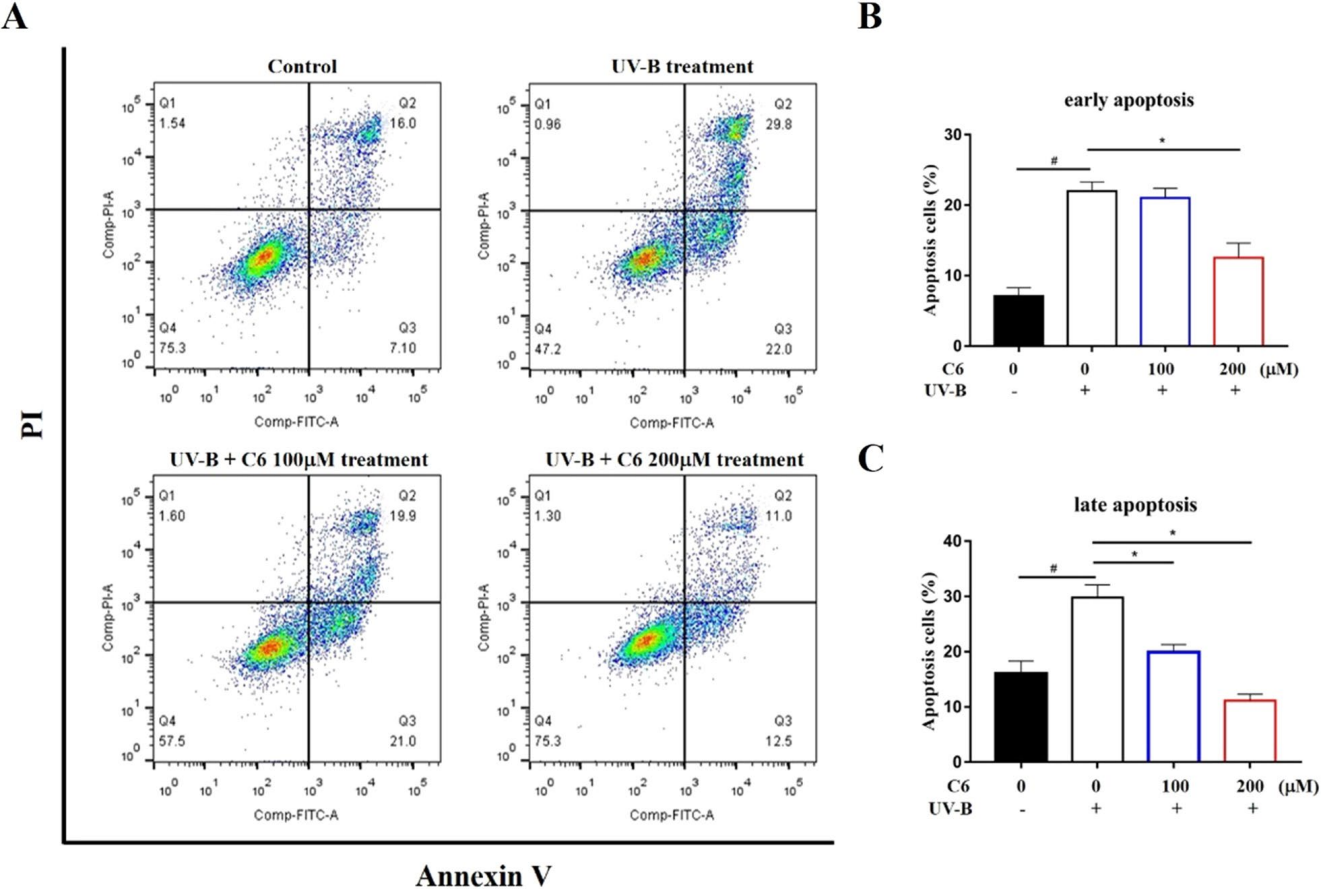
Bibenzyl derivatives in *D. officinale* reduce the cell apoptosis. **A** Typical flow charts determinate by Annexin V/PI double staining. The necrotic cells were identified as Annexin V−/PI+ (Q1), **C** late apoptotic cells as Annexin V+/PI+ (Q2), **B** early apoptotic cells as Annexin V+/PI− (Q3), and live cells as Annexin V−/PI− (Q4). ^#^P < 0.05, significantly different from control group; *P < 0.05, significantly different from model group

To evaluate the anti-apoptosis effect of bibenzyl derivatives in *D. officinale*, Annexin V/PI double staining was performed by flow cytometry. The exposure with UV-B irradiation in HaCaT cells induced 29.8% of early apoptotic cells and 22.0% of late apoptotic cells which was significantly higher than the control group (Fig. 3A). After treatment with different concentrations of C6, early apoptosis and late apoptosis were reduced in a dose-dependent manner (Fig. 3B, C). The above results show that bibenzyl derivatives can reduce apoptosis caused by UV-B irradiation.

### Bibenzyl derivatives reduces ROS accumulation in HaCaT cells

**Fig. 4:**
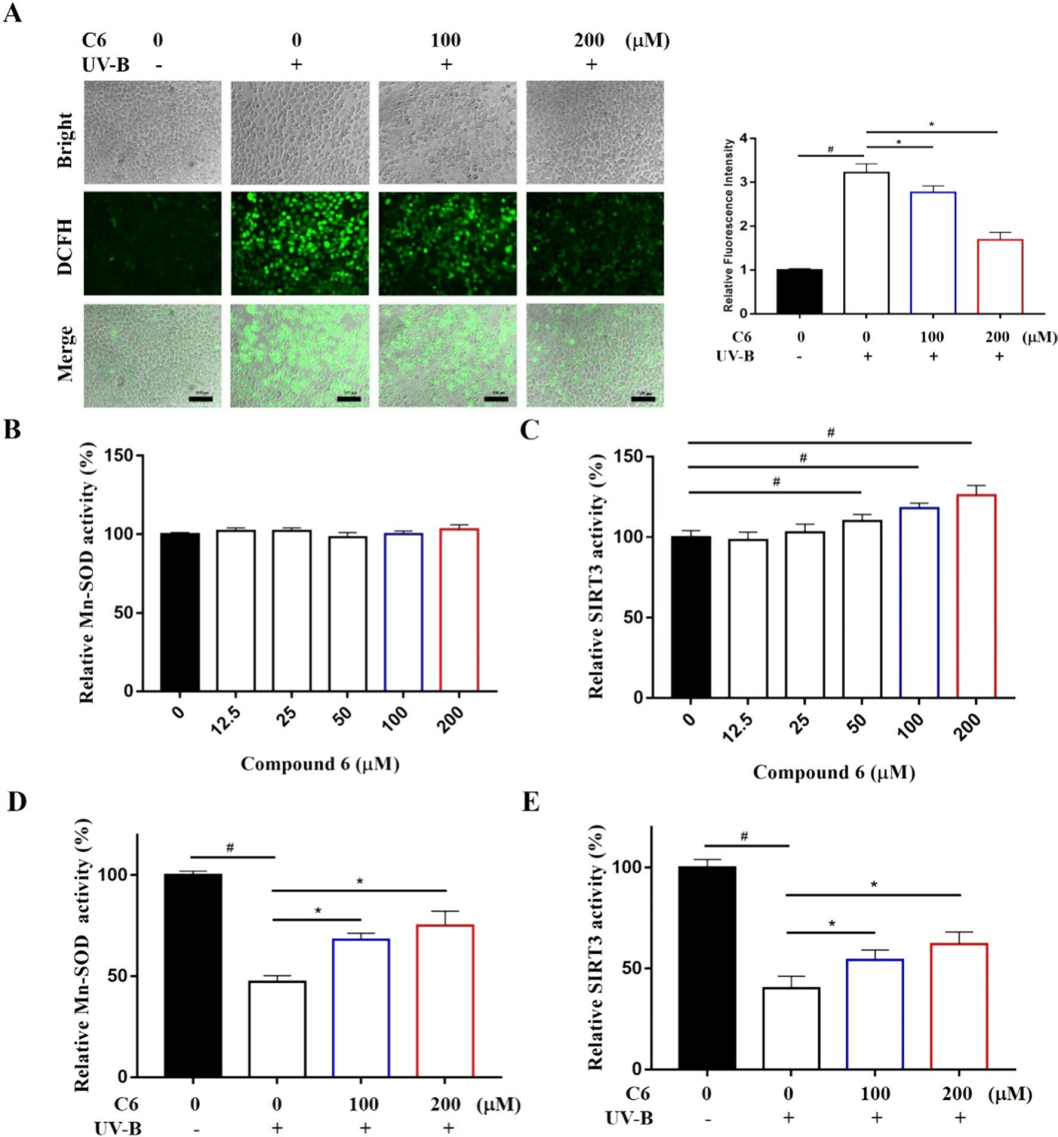
Study on the mechanism of reactive oxygen species scavenging properties of bibenzyl derivatives. **A** Intracellular ROS scavenging performance of C6 (magnification: ×20). **B** C6 did not directly enhance Mn-SOD activity in vitro. **C** C6 directly enhances SIRT3 activity. Effects of C6 on Mn-SOD (**D**) and SIRT3 (**E**) enzyme activities in HaCaT cells after UV-B irradiation. ^#^P < 0.05, significantly different from control group; *P < 0.05, significantly different from model group

To prove the reactive oxygen species (ROS) scavenging properties of bibenzyl derivatives in *D. officinale*, a 2′,7′-dichlorofluorescein diacetate (DCFH-DA) probe was used to assess the levels of intracellular ROS level. Take C6 as an example, the results of fluorescence staining showed that C6 could significantly reduce the accumulation of ROS in HaCaT cells induced by UV-B irradiation (Fig. 4A). Compared with the control group, the content of ROS in the cells increased significantly after UV-B irradiation. After the addition of C6, the content of intracellular ROS gradually decreased with the increase of concentration, indicating that bibenzyl derivatives can reduce the accumulation of intracellular ROS caused by UV-B irradiation.

### Effect of bibenzyl derivatives on related enzyme activities in vitro

Mn-SOD is a kind of superoxide dismutase located in the mitochondrial matrix which takes manganese ion as the active center, can protect the cells from oxidative stress by converting superoxide anion into less toxic hydrogen peroxide [23]. We further studied the effect of bibenzyl derivatives in *D. officinale* on Mn-SOD and SIRT3 activity in vitro. The addition of C6 in the Mn-SOD pure enzyme reaction system had no significant effect on Mn-SOD activity (Fig. 4B). As a deacetylation substrate of SIRT3, the activation of SIRT3 can promote the activity of Mn-SOD. Bibenzyl derivatives, taking C6 as an example, could significantly enhance the enzyme activity of SIRT3 (Fig. 4C). The above results show that bibenzyl derivatives has no direct effect on Mn-SOD activity in vitro, but can promote the enzyme activity of regulator SIRT3.

**Fig. 5:**
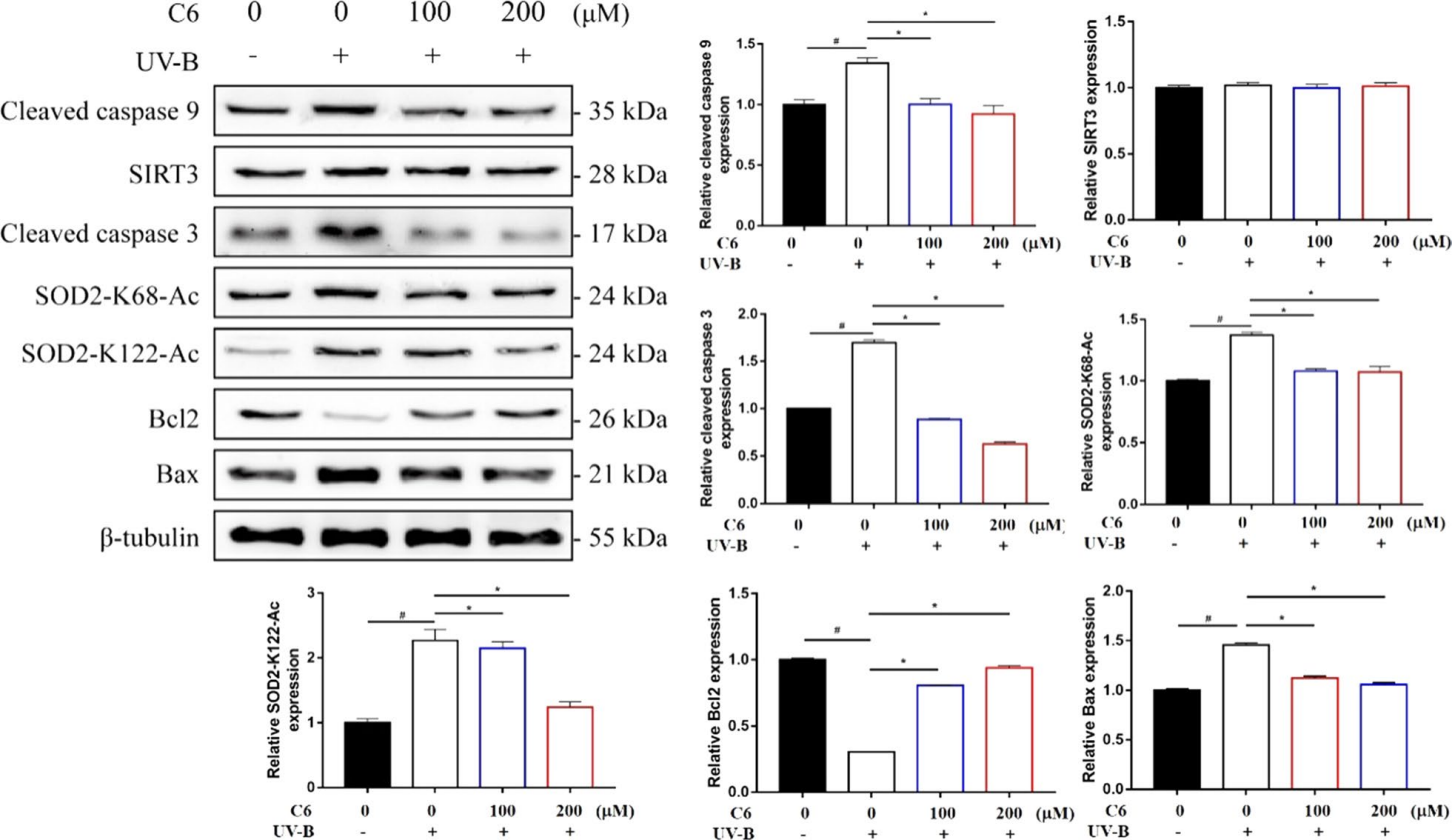
The changes of protein expression in HaCaT cells were detected by Western blot assay

We further detected the enzyme activities of Mn-SOD and SIRT3 in cell lysate by enzyme activity detection kit. After UV-B irradiation, the activities of Mn-SOD and SIRT3 in the model group decreased significantly. After treatment with different concentrations of C6, the activities of Mn-SOD and SIRT3 were significantly higher than those in the model group (Fig. 4D, E). The results of immunoblotting showed that UV-B irradiation significantly increased the acetylation level of Mn-SOD in the model group. The decrease of the protein expression levels of SOD2-K68-Ac and SOD2-K122-Ac represents the acetylation of Mn-SOD at two sites downregulated significantly after treated with different concentrations of C6. It should be noted that the application of bibenzyl derivatives had no effect on the protein expression level of SIRT3 (Fig. 5).

The above results suggest that bibenzyl derivatives in *D. officinale* may enhance Mn-SOD activity by promoting the deacetylation by SIRT3.

### Effect of bibenzyl derivatives on the expression of apoptosis related proteins in cells

The accumulation of ROS in HaCaT cells will promote the release of cytochrome C to the cytoplasm, activate the caspase cascade and cause cell apoptosis [24]. The results showed that UV-B irradiation significantly increased the activation of caspase 3 and caspase 9 and the expression of Bax in the model group, while the antiapoptotic protein Bcl-2 decreased significantly. After treated with different concentrations of C6, the activation of caspase 3 and caspase 9 and the expression of Bax were inhibited significantly, while the protein expression of Bcl-2 increased significantly (Fig. 5). The above results showed that the bibenzyl derivatives in *D. officinale* could reduce the apoptosis induced by UV-B irradiation.

## Discussion

At present, *Dendrobium officinale* as a valuable cosmetic raw material, has been widely used in the cosmetic industry [25]. However, the research on anti-aging activity of *D. officinale* mainly stays at the stage of crude extract, and there is a lack of systematic and in-depth research on the mechanism [26]. In the experiment, we found that bibenzyl derivatives in *D. officinale* had anti-photoaging effect, and then studied its mechanism.

In this study, an in vitro UV-B irradiation model was established to verify the anti-photoaging effect of bibenzyl derivatives. The results showed that bibenzyl derivatives could significantly scavenge ROS and inhibit the expression of pro apoptotic proteins in HaCaT cells irradiated by UV-B. In addition, bibenzyl derivatives could reverse the decrease of Mn-SOD and SIRT3 activities and the increase of Mn-SOD acetylation level in UV-B irradiated HaCaT cells. The molecular docking and surface plasmon resonance (SPR) results showed that bibenzyl derivatives directly bound to SIRT3 with high affinity. These results show that dibenzyl directly stimulates the SIRT3 activity, thereby reducing the acetylation level of Mn-SOD to enhance its antioxidant activity, and finally alleviate the cell damage caused by ROS accumulation caused by UV-B irradiation.

## Conclusion

This paper confirmed that, as the main composition of *Dendrobium officinale*, bibenzyl derivatives can protect cells from UV-B irradiation induced photoaging by activating SIRT3 activity. The results provide a theoretical reference for the exploration of skin care compositions and contribute to application of *D. officinale* in the development of domestic high-end cosmetics.

## Experimental

### Chemicals and reagents

**Fig. 6:**
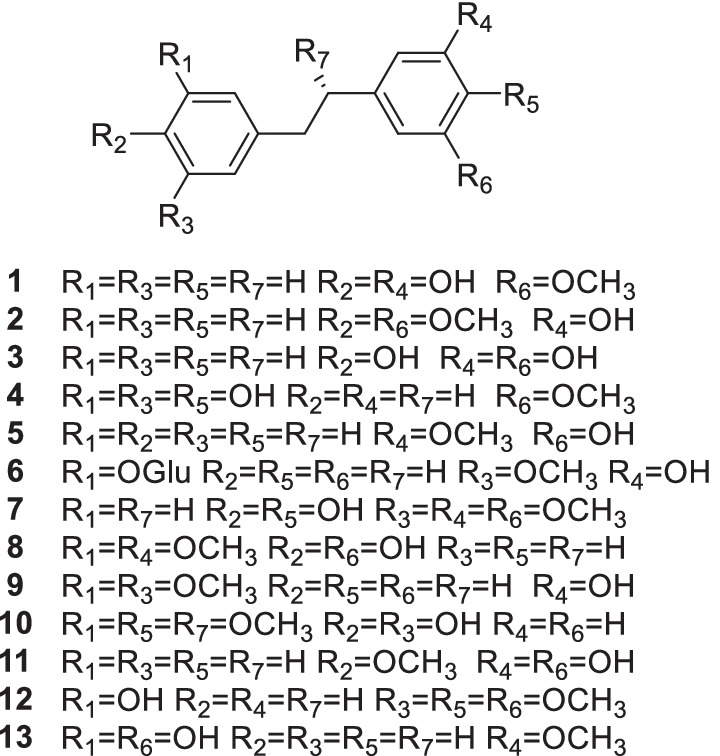
The structure of 13 bibenzyl derivatives in this study

13 bibenzyl derivatives from *D. officinale* were used in this study are extracted by our laboratory and the purities are as follows: **1** (99.72%), **2** (99.23%), **3** (95.33%), **4** (96.77%), **5** (99.42%), **6** (99.53%), **7** (99.32%), **8** (99.15%), **9** (99.54%), **10** (97.21%), **11** (99.12%), **12** (96.45%), **13** (98.64%) (Table 2, Fig. 6). DMEM (high glucose), fetal bovine serum and penicillin–streptomycin solution were purchased from Biological Industries (Kibbutz Beit Haemek, Isreal). Cell culture dishs were sourced from NEST (Wuxi, Jiangsu, CN). Trypsin (0.25%) solution were obtained from Cytiva (Little Chalfont, Buckinghamshire, UK). Reagents, glycine, tris-(hydroxymethyl)-aminomethane (Tris), sodium dodecyl sulfate (SDS) and cell lysis buffer for the Western and IP were of Meilunbio (Dalian, Shandong, CN). Proteintech (Wuhan, Hubei, CN) provided the primary antibodies against Bax (cat. no. 50599-2-Ig), Bcl2 (cat. no. 12789-1-AP), Caspase 9 (cat. no. 10380-1-AP), SIRT3 (cat. no. 10099-1-AP), β-tubulin (cat. no. 10094-1-AP) and the secondary antibody (horse radish peroxidase-conjugated goat anti-rabbit IgG) (cat. no. SA00001-2). Primary antibodies against cleaved caspase 3 (cat. no. 32042), SOD2-K68-Ac (cat. no. ab137037), SOD2-K122-Ac (cat. no. ab214675) were purchased from Abcam (Cambridge, UK).

**Table 2:** The information of 13 bibenzyl derivatives in this study

No.	Compounds name	CAS registry number
**1**	3,4′-Dihydroxy-5-methoxybibenzyl	67884-29-1
**2**	3-Methoxy-5-(4-methoxyphenethyl) phenol	365221-83-6
**3**	5-[2-(4-Hydroxyphenyl) ethyl]-1,3-benzenediol	58436-28-5
**4**	5-[2-(4-Hydroxy-3-methoxyphenyl) ethyl]-1,3-benzenediol	139101-67-0
**5**	3-Hydroxy-5-methoxybibenzyl	17635-59-5
**6**	3-[2-(3-Hydroxyphenyl) ethyl]-5-methoxyphenyl *β*-D-glucopyranoside	189302-75-8
**7**	4-[2-(4-Hydroxy-3-methoxyphenyl) ethyl]-2,6-dimethoxyphenol	108853-14-1
**8**	3′,4-Dihydroxy-3,5′-dimethoxybibenzyl	83088-28-2
**9**	3-[2-(3,5-Dimethoxyphenyl) ethyl] phenol	168281-05-8
**10**	3-Methoxy-5-[(1R)-1-methoxy-2-(4-methoxyphenyl) ethyl]-1,2-benzenediol	1104820-01-0
**11**	5-[2-(4-Methoxyphenyl) ethyl]-1,3-benzenediol	90332-29-9
**12**	3-[2-(3,4-Dimethoxyphenyl) ethyl]-5-methoxyphenol	135545-84-5
**13**	3,3′-Dihydroxy-5-methoxybibenzyl	56684-87-8

### Molecular docking

The molecular docking studies were carried out using grid-based ligand docking program by Maestro 11.9 molecular docking suite incorporated in the Schrodinger package (Schrodinger, Inc., NY, US) [27]. The crystal structure of human SIRT3 protein obtained by crystal X-ray diffraction was retrieved from Protein Data Bank (PDB ID: 4C7B, resolution: 2.10 Å) [28]. The preparation of bibenzyl derivatives structures were carried out by software ChemBioDraw Office 14.0 (CambridgeSoft, MA, US). In addition, optimized potential for liquid simulations (OPLS_2005) force field was applied for the structure of SIRT3 protein closest to the active receptor in the organisms [29]. After preparing ligands, proteins, and preparation of grid formation of the active site of protein, Glide docking suites output GScore (empirical scoring function) by predicting the best binding orientation to the protein target [30].

### Surface plasmon resonance measurement

Human SIRT3 synthetic peptide (cat. no.PEP-1085) was purchased from Thermo Fisher Scientific (Waltham, MA, US). The surface plasmon resonance (SPR) binding analysis were carried out using a Biacore S200 instrument (GE Healthcare, MA, US). The immobilization of SIRT3 protein on the surface of the Series S CM5 Sensor chip (GE Healthcare) was performed by the injection of protein solution (10 μg/mL) in sodium acetate buffer (10 mM, pH 5.0). The bibenzyl derivatives in *D. officinale* were dissolved in running buffer (PBS + 5% DMSO) and passed over the immobilized SIRT3 sensor surface at various concentrations (62.5–1000 μM, double dilution) at a flow rate of 30 μL/min. The binding time was 120 s, and the dissociation time was 150 s. Kinetics and affinity analyses were calculated based on bibenzyl derivatives at various concentrations using Biacore S200 Evaluation Software (GE Healthcare).

### Cell culture, UV-B irradiation model and viability assay

Human HaCaT cells were cultured routinely in DMEM (high glucose) supplemented with 10% fetal bovine serum and 1% penicillin–streptomycin solution at 37 °C in a humidified incubator at 37 °C and 5% CO_2_ [31]. All cells were cultured in culture dishes and the medium was changed every day. The cells were subcultured when the cell density reached 80%.

Briefly, The HaCaT cells were cultured for 24 h after plating at a density of 4 × 10^4^ cells/100 μL in 96-well plates. Subsequently, cells were irradiated according to the grouping using a 20 W UV-B lamp (SiTing, Shanghai, CN) at a distance of 10 cm for 5 s. The irradiation dose was calculated to be approximately 0.80 J/cm^2^. Cells were divided into the following groups: control, UV-B alone, UV-B + low dose bibenzyl derivatives (100 μM) and UV-B + high dose bibenzyl derivatives (200 μM). After UV-B irradiation, HaCaT cells continued to culture for 12 h with the medium containing different concentrations of bibenzyl derivatives. The effects of bibenzyl derivatives in *D. officinale* on cell protection was determined by the MTS assay (BestBio, Shanghai, CN). After incubation, cells each well were treated with 20 μL MTS solution for 1 h. The absorbance at 490 nm was measured directly with a microplate reader (FlexStation3, Molecular Devices, CA, US). Cell viability was expressed as the ratio percentage of MTS after deducting background value, assuming that the absorbance of control cells with deducting background absorbance was 100%.

### Annexin V/PI double staining

An Annexin V-FITC/PI Apoptosis Detection Kit (Yeasen, Shanghai, CN) was used to count the apoptotic cells. Briefly, The HaCaT cells were cultured for 24 h after plating at a density of 8 × 10^5^ cells/2 mL in 96-well plates. After treated according to groups, the HaCaT cells were resuspended in Annexin V/PI labeling solution for 10 min in the dark at room temperature then quantified immediately by flow cytometer (FACSCalibur, BD FACS Canto, USA).

### Reactive oxygen species detection

A reactive oxygen species assay kit (Nanjing Jiancheng Bioengineering Institute) was used to detect the intracellular reactive oxygen species (ROS) level in HaCaT cells [32]. The HaCaT cells were seeded in 24-well plates at a density of 2 × 10^5^ cells/500 μL. After treated according to groups, HaCaT cells were incubated in DMEM medium containing 10 μM 2′,7′-dichlorofluorescein diacetate (DCFH-DA) probe for 1 h to establish a stable intracellular level of the probe. The cells were washed with phosphate buffered saline (PBS) for five times and observed under a fluorescence microscope (DM5500B, Lecia, GER) at 20× magnification. DCFH-DA penetrating cells was initially transformed into dichlorofluorescin (DCFH) by intracellular related enzymes. Then DCFH was oxidized to dichlorofluorescein (DCF) in the presence of reactive oxygen species which showed green light under fluorescence microscope [33]. The intracellular ROS level was reflected by detecting the DCF fluorescence intensity.

### Mn-SOD activity in vitro

The superoxide dismutase typed assay kit (Nanjing Jiancheng Bioengineering Institute, Jiangsu, CN) was used to detect whether the bibenzyl derivatives in *D. officinale* had a direct promoting effect on Mn-SOD enzyme activity. Briefly, the cell samples were extracted with lysate buffer after the cells grew to 80% in one cell dish. Different concentrations of bibenzyl derivatives were added to the experimental wells, and the same volume of distilled water was added to the control wells to maintain a constant final volume. Finally, Mn-SOD enzyme activity was detected according to the manufacturer’s instructions.

Adherent HaCaT cells were incubated in a 6-well plate at the density of 1 × 10^6^ cells per well for 24 h. According to the method of UV-B irradiation model, the cells were treated with UV-B irradiation and bibenzyl derivatives. After 12 h, the total protein was extracted by cell lysis buffer. After the protein concentration of all samples is unified, the enzyme activity is detected according to the instruction of the assay kit.

### SIRT3 activity in vitro

SIRT3 direct fluorescent screening assay kit (Abnova, Taiwan, CN) was used to evaluate the effect of bibenzyl derivatives in *D. officinale* on SIRT3 enzyme activity. According to the manufacturer’s instructions, the activator wells contain 25 μL assay buffer, 5 μL diluted SIRT3 enzyme and 5 μL corresponding concentrations of bibenzyl derivatives were incubated with substrate solution for 45 min at 37 °C. Fluorescence was measured using a microplate reader at an excitation wavelength of 350 nm and an emission wavelength of 450 nm.

For the adherent HaCaT cells, the experimental method was consistent with the above-mentioned Mn-SOD assay after the total protein was taken for detection.

### Western blot

Cell lysis was carried out on ice for 30 min by cell lysis buffer containing 1% phenylmethylsulfonyl fluoride (Beyotime Biotechnology, Shanghai, CN). After centrifugation at 15,000 rpm for 5 min, the lysate was collected and stored at − 70 °C. The protein concentration was quantified using a total protein assay kit (Nanjing Jiancheng Bioengineering Institute). 50 μg protein samples were electrophoresed with loading buffer on a 12% sodium dodecyl sulfate polyacrylamide gel. Proteins were transferred to polyvinylidene difluoride membranes (Millipore, MA, US) at 200 mA for 1 h. The blots were incubated with corresponding primary antibodies diluent and secondary antibodies diluent successively after blocking in blocking buffer (0.1% Tween-20 in tris-buffered saline, containing 5% non-fat milk powder) at room temperature for 1 h. The bands of interest were incubated with enhanced chemiluminescent substrate solution (Proteintech).

### Data analysis

All data are presented as mean ± standard deviation of at least three independent experiments. Experimental values between groups were evaluated by one-way analysis of variance. A value of P less than 0.05 was considered to be statistically significant. Data were analyzed and plotted using Prism 7.0 software (GraphPad, CA, United States).
